# Combined antiresorptive and new anabolic drug approach in osteogenesis imperfecta zebrafish models

**DOI:** 10.1093/jbmrpl/ziaf112

**Published:** 2025-07-02

**Authors:** Cecilia Masiero, Francesca Tonelli, Carla Aresi, Marta Filibian, Daria Larionova, Silvia Cotti, Filippo Doria, Camilla Torriani, Paola Bertuccio, Anna Odone, Simona Villani, Antonio Rossi, Paul Eckhard Witten, Antonella Forlino

**Affiliations:** Department of Molecular Medicine, Biochemistry Unit, University of Pavia, 27100 Pavia, Italy; Department of Molecular Medicine, Biochemistry Unit, University of Pavia, 27100 Pavia, Italy; Department of Molecular Medicine, Biochemistry Unit, University of Pavia, 27100 Pavia, Italy; Centro Grandi Strumenti, University of Pavia, 27100 Pavia, Italy; Evolutionary Developmental Biology Group, Department of Biology, Ghent University, 9000 Ghent, Belgium; Department of Molecular Medicine, Biochemistry Unit, University of Pavia, 27100 Pavia, Italy; Department of Chemistry, University of Pavia, 27100 Pavia, Italy; Department of Public Health and Experimental and Forensic Medicine, Unit of Biostatistics and Clinical Epidemiology, University of Pavia, 27100 Pavia, Italy; Department of Public Health and Experimental and Forensic Medicine, Unit of Hygiene, University of Pavia, 27100 Pavia, Italy; Department of Public Health and Experimental and Forensic Medicine, Unit of Hygiene, University of Pavia, 27100 Pavia, Italy; Department of Public Health and Experimental and Forensic Medicine, Unit of Biostatistics and Clinical Epidemiology, University of Pavia, 27100 Pavia, Italy; Department of Molecular Medicine, Biochemistry Unit, University of Pavia, 27100 Pavia, Italy; Evolutionary Developmental Biology Group, Department of Biology, Ghent University, 9000 Ghent, Belgium; Department of Molecular Medicine, Biochemistry Unit, University of Pavia, 27100 Pavia, Italy

**Keywords:** osteogenesis imperfecta, zebrafish, skeleton, chemical chaperone, bisphosphonate, osteoblasts, osteoclasts, osteocytes, endoplasmic reticulum stress

## Abstract

Osteogenesis imperfecta (OI) is a family of heritable collagen I–related skeletal disorders for which, to date, no definitive cure is available. Individuals with OI are mainly treated with bisphosphonates that enhance bone mass by inhibiting bone resorption. However, new strategies combining antiresorptive molecules with bone anabolic drugs are likely to provide valid alternatives for skeletal health, protecting physiological bone turnover. Recently, cellular stress has been identified as a therapeutic target in both dominant and recessive forms of OI characterized by overmodified collagen I. The chemical chaperone 4-phenylbutyrate (4PBA) successfully ameliorated cell homeostasis in both in vitro and in vivo OI models. In this study, dominant *Chihuahua* (*Chi/+*) and recessive *p3h1^−/−^* zebrafish OI models were treated for 2 mo either with the bisphosphonate alendronate (ALN) or with 4PBA or with a combination of the two. The treatment effect at the tissue level was evaluated by microCT analysis of the vertebral body, while histology and gene expression analyses allowed to dissect the consequences at a cellular level. Only ALN administration improved the vertebral thickness in the dominant *Chi/+* model. The combined therapy synergistically improved osteoblast homeostasis and promoted the formation of mature extracellular collagen fibers in both models. All treatment conditions reduced osteoclast TRAP activity in *Chi/+*, whereas 4PBA and 4PBA + ALN had the opposite effect on *p3h1^−/−^*. Finally, 4PBA and the combination of ALN and 4PBA reduced osteocyte apoptosis only in *p3h1^−/−^*. Our data demonstrated for the first time in vivo a differential effect of the combination of an antiresorptive and a new anabolic compound in dominant and recessive OI zebrafish models, stressing the importance of identifying the specific causative molecular defect to define the best treatment option.

## Graphical Abstract

**Graphical Abstract:**
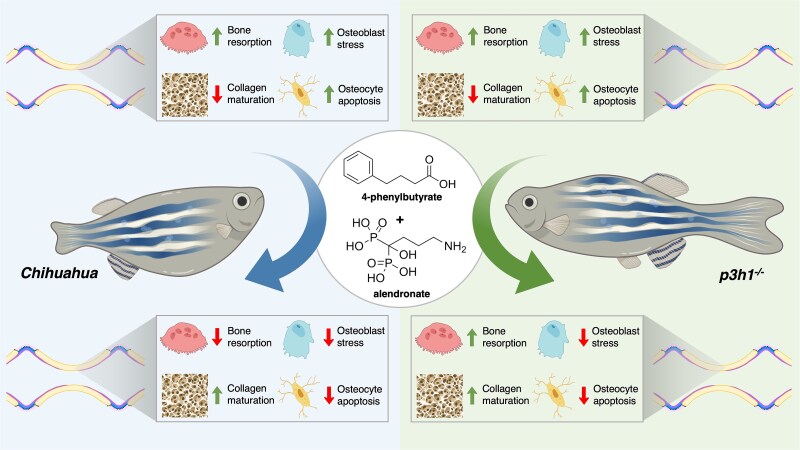

## Introduction

Osteogenesis imperfecta (OI) is a rare heterogenic group of genetic skeletal dysplasia characterized by bone fragility, skeletal deformities, frequent fractures, and vertebral compressions.^1^ The most common forms of the disease are caused by dominant mutations in *COL1A1* and *COL1A2* genes, encoding proα1 and proα2 chains of collagen I, respectively. The most frequent mutation is the substitution of a glycine within the conserved Gly-Xaa-Yaa triple helix motif, essential for proper collagen folding.^1^ Glycine replacement with a bulkier or charged amino acid causes collagen I structure alteration and folding delay.^2^ Since 2006, new mutations in collagen-related genes, mainly characterized by recessive inheritance, have been described as responsible for OI.^1^ Among others, OI type VIII is triggered by defects in prolyl 3-hydroxylase 1 (P3H1), a member of the endoplasmic reticulum (ER) resident prolyl 3-hydroxylation complex that is involved in the 3-hydroxylation of the α1(I)-Pro986 and in supporting collagen I triple helix folding and assembly.^1^ Alterations in collagen I structure as well as 3-hydroxylation complex impairment lead to collagen I retention inside the ER. This ER retention causes collagen I to undergo excessive post-translational modifications. As a result, collagen accumulation and over-modification within the ER result in cellular stress.^3^ Furthermore, affected procollagen molecules secreted in the extracellular matrix negatively impact fibril assembly, structures, and interactions with other non-collagenous proteins.^2^

In recent years, the zebrafish (*Danio rerio*) has emerged as a valuable non-mammalian vertebrate model for studying skeletal diseases, due to its conserved skeletal structures, bone-specific gene expression profile, bone cell types, and ossification processes shared with mammals.^4^ Moreover, the zebrafish offers several technical and cost-related advantages over the more commonly used rodent models, making it a valuable tool for the in vitro/in vivo translation of drug screening.^4^ Given its involvement in human skeletal diseases, the vertebral column is one of the most studied structures in the biomedical field. The similarities between the axial skeleton of teleosts and mammals, such as physiological curvatures and the conservation of mechanical load response, make zebrafish a valuable model for studying bone formation and testing new drugs. However, it is important to note that, while load response is conserved, its origin differs: in zebrafish, it results from swimming-generated forces, whereas in terrestrial vertebrates, it is primarily driven by gravity. Additionally, the presence of various zebrafish mutants with vertebral column deformities further amplifies their value in skeletal research.^5^ With the use of forward and reverse genetic approaches, several zebrafish models that recapitulate specific diseases, such as OI, have been generated.^5^ Among others, *Chihuahua* (*Chi/+*) carries a p.(Gly736Asp) substitution in the α1 chain of collagen I and resembles severe dominant OI, while *p3h1^−/−^* zebrafish, generated by CRISPR/Cas9 gene editing, carries a p.(Asp196Glufs*266) in *p3h1* and recapitulates the severe OI type VIII phenotype. Both models share an altered skeletal development starting from the larval stage that worsens in adulthood, when they are characterized by impaired bone geometrical parameters, vertebral body fusions, calli, deformities, and fractures.^6–9^ Collagen I extracted from *Chi/+* and *p3h1^−/−^* tissue is overmodified and partially retained inside the ER, leading to cisternae enlargement.^8^^,^^9^

No definitive cure is available for OI and the most common treatment is with bisphosphonates (BPs), anticatabolic molecules that block osteoclast (OC) activity and bone remodeling.^10^ Recently, the positive role of 4-phenylbutyrate (4PBA), a Food and Drug Administration (FDA)–approved drug for urea cycle disorders,^11^ was demonstrated in OI models both in vitro and in vivo.^3^^,^^7^^,^^12–15^ 4PBA improved general protein secretion, with positive effects on osteoblast homeostasis, collagen secretion, and matrix mineralization.^13^ Its administration in dominant OI *Aga2^+/−^* mice, characterized by a c.-16 T>A frameshift mutation in exon 50 of *Col1a1*, increased femoral cortical thickness and decreased fracture incidence.^15^ 4PBA administration favored collagen secretion and improved bone mineralization in *Chi/+* zebrafish and improved *p3h1^−/−^* caudal fin ray regeneration ability.^7^^,^^14^

Our study aimed to investigate the anticatabolic activity of alendronate (ALN), the anabolic effect of 4PBA, and the combination of the two specifically on bone using *Chi/+* and *p3h1^−/−^* zebrafish models. Bone geometrical properties of the caudal vertebral column were evaluated by micro-CT (μCT), while quantitative PCR (qPCR) and histology allowed to dissect the role of single and combined treatments at a cellular level. We demonstrated a major effect of ALN in the amelioration of bone geometrical parameters in *Chi/+* zebrafish. The positive effect of the combined therapy was more evident at a cellular level, as demonstrated by improved cell homeostasis and increased collagen I fiber maturation, suggesting the need for a longer treatment duration to translate the effect from cells to tissue. The detection of a mutation-specific effect of the pharmacological regimens suggests the need for a personalized medical approach for the effective treatment of OI.

## Materials and methods

### Animal husbandry

Wild-type AB zebrafish (WT) were obtained by the European Zebrafish Research Center (EZRC, Germany). Osteogenesis imperfecta mutants *Chihuahua* (*col1a1a^dc124/+^, Chi/+*)^16^ and *p3h1^upv2/+^(p3h1^−/−^*)^9^ were used. Adult zebrafish were housed in the animal facility of the University of Pavia in a ZebTEC system (Tecniplast, Buguggiate, Italy) at 28 °C, pH 7.5, and conductivity of 500 μS on a 14/10 light/dark cycle and fed 3 times per day—alternating dry food and brine shrimps. Developing embryos were kept in petri dishes in E3 medium (NaHCO_3_ 1.2 mM, instant ocean 0.01% wt/vol, CaSO_4_ 1.4 mM, methylene blue 0.00002% wt/vol) at 28 °C. The WT and *Chi/+* genotypes were obtained as previously described.^7^ For the experiments, embryos and adult fish were anesthetized using a solution of tricaine (3-amino benzoic acidethylester; Merck, Darmstadt, Germany) 0.016% wt/vol in E3 medium or system water, respectively and killed by tricaine overdose (0.03% wt/vol).

All the experiments complied with the EU Directive 2010/63/EU and were approved by the Italian Ministry of Health (260/2020-PR). A detailed description of the fish groups used in this study is provided in Table S1.

### Alendronate toxicity test

The WT embryos were manually dechorionated at 24 h post-fertilization (hpf), placed in 6-well plates (10 embryos per well), and treated for 7 d with 10, 20, or 30 μM ALN (purity 97%, synthetized in house). A group of untreated embryos kept in E3 medium was used as a negative control. Half of the volume of the solution with or without ALN was replaced every day. The numbers of surviving and misshaped embryos were daily evaluated until the end of the treatment according to Organization for Economic Co-operation and Development (OECD) guidelines.^17^

### 4PBA and ALN treatments

The *Chi/+* embryos were manually dechorionated at 24 hpf, placed in 6-well plates (20 fish per well), and treated with 50 μM 4PBA (Merck, Darmstadt, Germany) and/or 30 μM ALN dissolved in E3 medium until 11 d post-fertilization (dpf) according to the following strategy: during the first 3 d embryos received only 4PBA, from day 4 to day 8 4PBA was combined with ALN, while from day 9 to day 11 embryos received only 4PBA (Figure S1A). Half of the volume of the solution was daily replaced. At the end of the treatment, larvae were sacrificed and fixed in 4% paraformaldehyde (PFA).

Adult *Chi/+* and WT siblings and, in order to optimize the number of fish with a specific genotype, *p3h1^−/−^* and WT zebrafish obtained by different matings were treated for 2 mo (from 6 to 8 mo post-fertilization [mpf]) with 50 μM 4PBA, dissolved in system water and changed daily (4PBA-only) or 30 μM ALN injected intraperitoneally (ALN-only) or with the combination of both drugs (4PBA + ALN) (Figure S1B). For the injection, 5 μL ALN dissolved in Danieau solution (NaCl 58 mM, KCl 0.7 mM, MgSO_4_ 0.4 mM, Ca(NO3)_2_ 0.6 mM, Hepes 5 mM, pH 7.6) containing a tracer dye (dextran conjugated with tetramethyl rhodamine 0.5 mg/mL; Molecular Probes, Carlsbad, CA, USA) was used. A placebo group injected only with Danieau solution and tracer dye and kept in regular system water was added to each experiment. At the end of treatment, fish were sacrificed and collected for the following described experiments.

### Whole-mount skeletal staining

To evaluate the effect of the treatments on larvae mineralization, Alizarin red S staining was performed, as previously described.^7^^,^^9^ Images were acquired using a Leica (Wetzlar, Germany) M165 FC microscope connected to a Leica DFC425 C digital camera. The notochord (NC), cleithrum (CL), hyomandibular (HM), and ceratohyal (CH) bone mineralization was evaluated. Three operators blinded to treatment and genotype classified the levels of ossification as incomplete or complete, as previously described.^7^^,^^9^

### Micro-CT

The fish caudal part of the body from adult *Chi/+* and WT siblings and *p3h1^−/−^* and WT zebrafish obtained by different matings was collected, fixed overnight at 4 °C in 4% PFA (wt/vol), and bone geometrical parameters acquired by μCT. Acquisition was performed using the Skyscan 1276 (Bruker, Kontich, Belgium). Scans were acquired at 55 kV and 72 μA, with a 0.25-mm aluminum filter and at an isotropic voxel size of 4 μm. Ring artifact suppression was active during acquisitions. Reconstructions were performed with NRecon software (Bruker), maintaining smoothing and beam hardening correction constant. Reconstructed cross-sections were rotated in DataViewer (Bruker) to lie along the long axis of the vertebra normal to the transaxial plane, and an ROI was drawn (Figure S2). A fixed threshold was used to segment the vertebral body. The bone volume (BV; mm^3^) and vertebral thickness (V.Th; mm) were evaluated using CTAn software (Bruker). The 2D V.Th along the *z* axis (the long axis of the vertebra) was also evaluated in regions identified on the basis of the maximum shrinkage point and abdominal (aEP) or caudal (cEP) endplates. Due to the differences between WT and mutants’ vertebral length, the minimum of the V.Th curve (ie, the centrum) of each vertebra was shifted to the origin of the *z* coordinate. An 84-μm region was established to compare vertebrae centra of both mutants, while 120- or 164-μm regions were evaluated in *Chi/+* or *p3h1^−/−^* vertebral endplates, respectively. The BMD (g/cm^3^) of the vertebral body was calculated using a 2-mm phantom rod pair containing 0.25 and 0.75 g/cm^3^ Calcium Hydroxyapatite (Skyscan).

### Geometric morphometrics of vertebral centra

The μCT-reconstructed vertebral column specimens were oriented and used to determine the shape of the first 10 caudal vertebral centra^18^ in WT and treated and untreated mutants, as previously described.^19^ Briefly, 10 landmarks were established at the endplates, anterior and posterior cone of the centrum, and at the maximum shrinkage region of the vertebra centrum. The 2D landmarks were extracted from DataViewer software (Bruker) and digitized maintaining the same order for all analyzed vertebral centra. Procrustes superimposition of digitized landmarks and visualization of shape variations were performed using Past4.04 software.

### Histology

Adult WT (siblings of *Chi/+*), *Chi/+*, and *p3h1^−/−^* zebrafish were collected after treatment for histology. The caudal part of the body was fixed in 4% PFA overnight at 4 °C and decalcified in 4% PFA, 10% EDTA in PBS, pH 7.4 for 3 wk at 4 °C, by changing the solution 3 times per week. Specimens were embedded in glycol methacrylate (GMA).^19^

To analyze vertebrae morphology and evaluate osteocyte number, 2-μm sections were stained with 0.5% toluidine blue, 1% Na_2_B_4_O_7_ in dH_2_O, pH 9 for 10 s, dried and mounted with dibutylphthalate polystyrene xylene. Slides were then digitalized with a MoticEasyScan infinity system (Motic, Kowloon, Hong Kong) and pictures were acquired with an Axio Imager-Z1 microscope (Carl Zeiss, Oberkochen, Germany) equipped with a 5-megapixel (5MP) charge-coupled device (CCD) camera. Intervertebral disc (IVD) morphology was evaluated on slide scans, distinguishing healthy and degenerated IVDs based on the alterations in IVD structure and organization (loss of structural integrity, IVD enlargement and dislocation). The percentage of healthy or degenerated IVDs on the total IVD number was calculated. At least 5 sections for each vertebra were evaluated.

Osteocytes were counted on the middle plane of caudal vertebrae sections using Fiji (ImageJ) software. Osteocyte number was normalized to the area of each respective analyzed vertebra.

Collagen fiber maturity was determined on 5-μm slides stained using 0.1% wt/vol Sirius Red (Direct Red 80; Merck) in saturated aqueous solution of picric acid (Merck) for 72 h at room temperature protected from light. Images were acquired both in bright-field and under polarized light using a Leica DM6B Wide Field microscope (Leica). Quantification of mature/red and immature/yellow-green collagen fibers was performed by normalizing stained collagen fibers’ area acquired on polarized light images to the total collagen area acquired in bright field.

### Tartrate-resistant acid phosphatase activity

To evaluate osteoclast (OC) activity, tartrate-resistant acid phosphatase (TRAP) staining was performed on 5-μm sections of WT (siblings of *Chi/+*) and treated and untreated mutant fish, as previously described.^19^ Slides were scanned with a MoticEasyScan infinity machine (Motic), and images were acquired using a Axio Imager-Z1 microscope (Carl Zeiss) equipped with an Axiocam 503 color camera (Carl Zeiss). The percentage of TRAP-positive vertebrae was then evaluated over the total number of analyzed vertebrae at the level of neural and haemal arches and on the autocentrum of caudal vertebrae.

### Transmission electron microscopy

Vertebral columns were dissected and fixed in paraformaldehyde-glutaraldehyde (PG) fixative (0.1 M sodium cacodylate, pH 7.4; 1.5% vol/vol glutaraldehyde; 1.5% wt/vol paraformaldehyde; 0.001% vol/vol CaCl_2_), as previously described.^20^ Decalcification was performed in PG fixative, 0.1 M EDTA for 4 wk by changing the solution twice a week. Samples were postfixed with osmium tetroxide (OsO_4_) and subsequently embedded in Epon epoxy resin. One-micrometer sagittal semi-thin sections and 70-nm ultrathin sections, displaying the middle plane of the vertebral column, were obtained using a Reichert-Jung Ultracut E ultramicrotome (C. Reichert Optische Werke AG, Wien, Austria). Ultrathin sections were contrasted with uranyl acetate and lead citrate. Sections were analyzed with a Jeol JEM 1010 transmission electron microscope (Jeol Ltd, Tokyo, Japan) operating at 60 kV, and microphotographs were acquired through a Veleta camera (Emsis, Muenster, Germany). Sagittal and transverse sections of the ER cisternae width were measured adapting the protocol previously described.^21^ ER cisternae thickness was measured every 50 nm on sagittal sections, while 8 to 10 measurements were performed on each transverse cisterna, using ImageJ (Fiji) software.

### TUNEL assay

To evaluate osteocyte apoptosis, a Click-iT Plus TUNEL assay (C10619; Invitrogen, Waltham, MA, USA) was performed on 5-μm sections of WT (siblings of *Chi/+*) and treated and untreated mutant fish following the manufacturer’s instructions. Positive and negative controls were prepared by treating samples with 1 unit of DNAse I (ThermoFisher, Waltham, MA, USA) in 1× reaction buffer for 30 min or without performing terminal deoxynucleotidyl transferase enzyme incubation, respectively (Figure S3). Images were acquired using a Leica DM6B Wide Field microscope (Leica). The number of DAPI-positive and TUNEL-positive cells inside the vertebrae autocentrum was counted using the Leica Application Suite X (LAS X) software (Leica). TUNEL-positive osteocyte numbers were standardized to the total number of osteocytes.

### Real-time qPCR

RNA was extracted from skulls and precaudal vertebrae of *Chi/+* and WT siblings and *p3h1^−/−^* and WT zebrafish obtained by different matings (a minimum of 3 replicates per group of genotype and treatment) using QIAzol lysis reagent (Qiagen, Hilden, Germany), according to the manufacturer’s instructions. cDNA was synthesized with the High-Capacity cDNA transcription kit (Applied Biosystems, Waltham, MA, USA). The expression of *collagen 1 a1a chain (col1a1a), osteocalcin (bglap), cathepsin K (ctsk), acid phosphatase 5a, tartrate resistant (acp5a), receptor Activator of Nuclear factor κ B Ligand (rankl),* and *osteoprotegerin (*opg)** was evaluated by reverse transcriptase qPCR (RT-qPCR)*. Loopern4* was used as the housekeeping gene. The relative expression was calculated using the ΔΔCt method. Primer sequences are reported in Table S2.

### Statistical analysis

Quantitative variables are expressed as mean ± SD, while qualitative variables used percentages. Normal distribution was tested using the D’Agostino and Pearson test. Grubbs’ test (alpha set at .05) was performed to identify outliers. Single comparisons between WT and untreated mutants were performed using unpaired *t* test, while to evaluate the effect of treatments on mutants, multiple comparisons were performed using 1-way ANOVA followed by Bartlett’s post hoc test when normality passed, or Kruskal-Wallis test when normality failed. Statistical analyses were performed using GraphPad Prism 9.3.1. To analyze Alizarin red data on larvae and IVD structure, Fisher’s exact test was used. *p* < .05 was considered significant.

To assess vertebral thickness trend variations between specific genotype and treatment groups, the Mann-Whitney *U* test was conducted, with a multiple-comparisons test if the *p* value was less than .05. Bonferroni’s correction for multiple comparisons was applied. Statistical analyses for both larvae mineralization and vertebral thickness trend variation were performed using R software version 4.4.1.

## Results

### Anabolic and/or antiresorptive therapy differently improved bone geometrical parameters in dominant and recessive OI zebrafish models

The combined anabolic and antiresorptive effect of 4PBA and ALN, respectively, as well as the response to single treatments (4PBA-only and ALN-only) were evaluated in both dominant and recessive forms of OI by exploiting larvae and adult *Chihuahua* (*Chi/+*)^7^ and *p3h1^−/−^* knockout^9^ zebrafish models, respectively.

Alendronate toxicity was first evaluated in WT zebrafish by administering the drug at 10, 20, and 30 μM from 1 to 7 dpf. All of the tested concentrations caused 100% lethality at day 7, whereas up to 5 dpf, the highest dose tested showed only 20% mortality rate and no deformity. Therefore, 30 μM ALN was selected limiting the treatment to 5 d (Figure S4A).

To investigate the potential effect of the treatments, an explorative experiment was performed using *Chi/+* larvae that were treated from 1 to 11 dpf and the mineralization level of their cranial bone was evaluated as previously described.^7^ To this aim, *Chi/+* larvae were incubated in fish water under different treatment conditions: 4PBA alone for the entire period (1 to 11 dpf); ALN alone, added from day 4 to day 8; or a combination of both compounds, with 4PBA administered from day 1 to 11 and ALN from day 8 to 11, as detailed in Figure S1A. An untreated group kept in E3 medium for all the period was used as a placebo. An increased level of complete ossification of the cleithrum (CL) was observed in larvae treated with the combination of 4PBA + ALN in comparison to untreated or 4PBA-only and ALN-only treated fish. A significant improved notochord (NC) ossification was detected in 4PBA + ALN treated larvae, but only compared with the ALN-only treated group (Figure S4B, C).

Based on these preliminary results obtained on larvae, the potential effect of the combined anabolic and anticatabolic effect (4PBA + ALN) on bone was further tested on adult fish. Therefore, a 2-mo treatment using 4PBA-only, ALN-only, and 4PBA + ALN was performed, as described in the Materials and methods section, on both dominant (*Chi*/+) and recessive (*p3h1^−/−^*) OI zebrafish models.^8^^,^^9^

**Figure 1:**
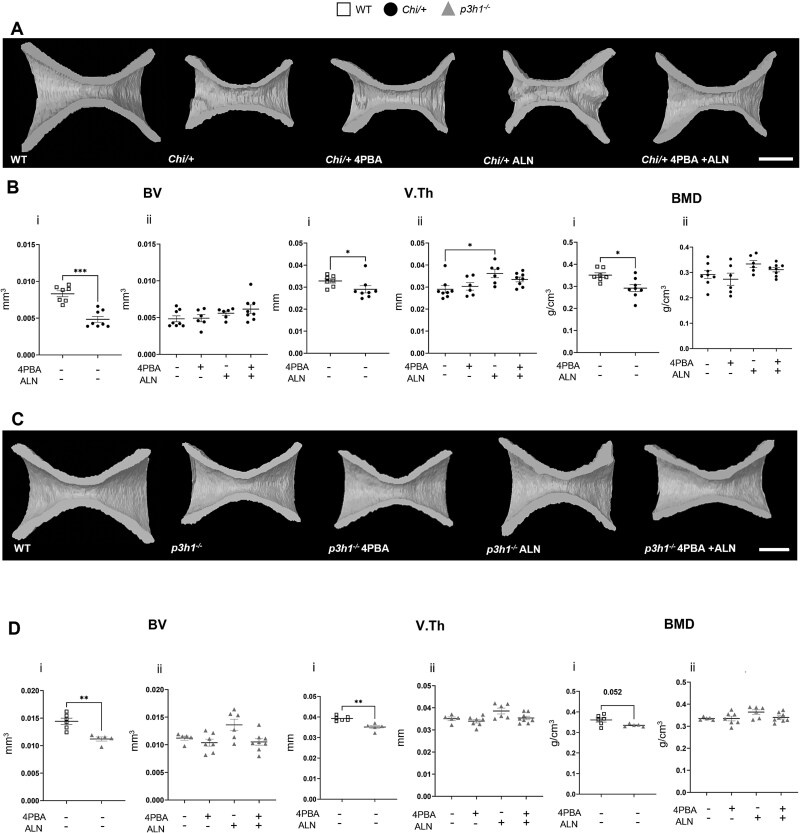
Bone geometrical parameters. (A) Representative 3-dimensional (3D) models of WT and untreated and treated *Chi/+* fish. Scale bar: 100 μm. (B) Micro-CT (μCT) analysis of bone volume (BV), vertebral thickness (V.Th), and BMD in WT, *Chi/+* (i), and *Chi/+* untreated and treated with 4PBA, ALN, and 4PBA + ALN (ii). (C) Representative 3D models of WT and untreated and treated *p3h1^−/−^* fish. Scale bar: 100 μm. (D) μCT analysis of BV, V.Th, and BMD in WT, *p3h1^−/−^* (i), and *p3h1^−/−^* untreated and treated with 4PBA, ALN, and 4PBA + ALN (ii). In the graphs, each dot represents a single value. ^*^p < .05, ^**^p < .01, ^*^^*^^*^p < .001. Abbreviations: ALN, alendronate; 4PBA, 4-phenylbutyrate.

At the end of the treatment the geometrical parameters of caudal vertebrae were evaluated by μCT.^6^^,^^9^ As expected, in *Chi/+* BV and V.Th were significantly reduced compared with WT fish and also BMD was lower in mutant fish (Figure 1A, B i). Only ALN significantly ameliorated *Chi/+* vertebral thickness compared with untreated *Chi/+* zebrafish (Figure 1A, B ii). In the *p3h1^−/−^*model, a reduction in BV and V.Th was observed compared with WT, while BMD impairment approached significance (*p* = .052) (Figure 1C, D i). No significant effect of the treatments was observed in the recessive OI model (Figure 1C, D ii). None of the treatments had an effect on WT fish (Figure S5). The collected data suggest that a 2-mo treatment in adult mutant fish improves vertebral thickness only when ALN is administered, and this effect is observed exclusively in the dominant OI zebrafish model.

### Vertebral thickness profile and vertebral shape are differently affected by 4PBA and/or ALN in dominant and recessive OI zebrafish models

**Figure 2:**
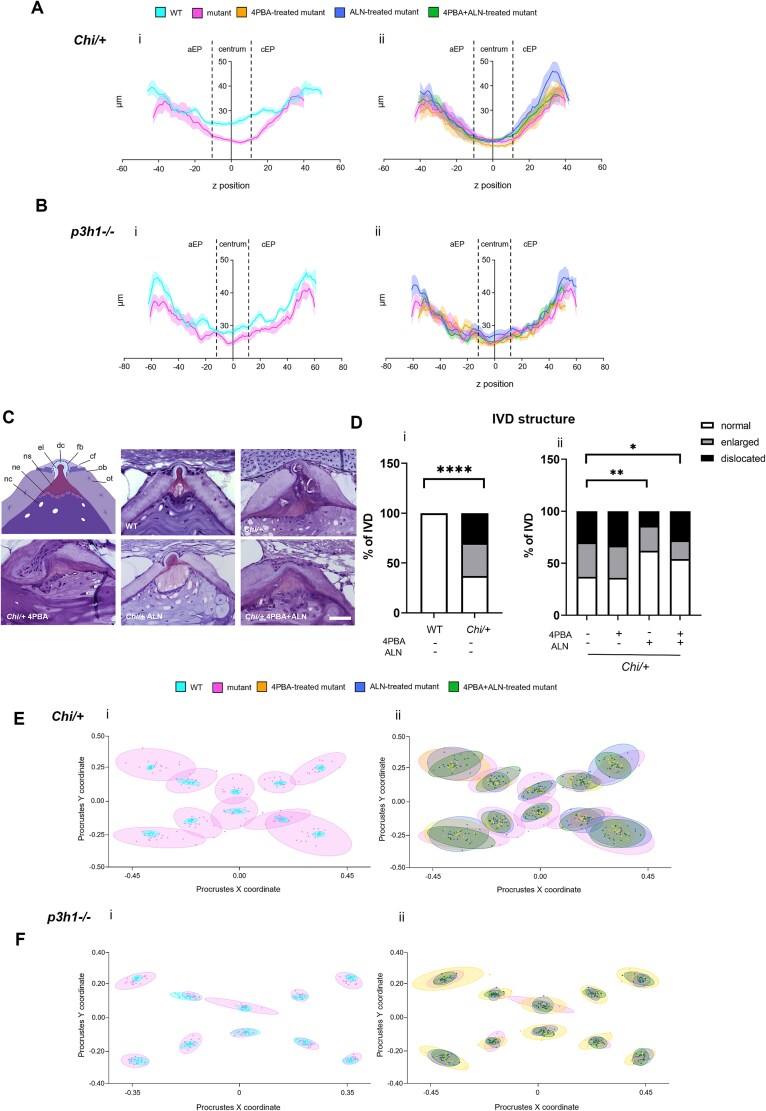
Two-dimensional (2D) analysis of vertebral thickness and intervertebral disk (IVD) structure in *Chi/+* and *p3h1^−/−^* zebrafish. (A, B) 2D vertebral thickness (V.Th) distribution along the *z*-axis of WT and untreated mutants (i) and between untreated and treated mutants (ii). Dashed lines outline the vertebral centrum and abdominal (aEP) or caudal (cEP) endplates. (C) IVD structure drawing displaying vertebral endplate elements and toluidine blue–stained sections showing WT and treated and untreated *Chi/+*. Scale bar: 40 μm. (D) IVD structure qualitative evaluation in WT, *Chi/+* (i), and *chi/+* untreated and treated with 4PBA, ALN, and 4PBA + ALN (ii). In the graph, the percentage of normal, enlarged, or dislocated IVDs on total number of analyzed IVDs is reported (^*^p < .05, ^**^p < .01, ^****^p < .0001). (E, F) 2D landmark scatterplot of WT and untreated mutant vertebrae (i) and untreated and treated mutant vertebrae (ii). Each dot represents a single landmark. Abbreviations: ALN, alendronate; cf, collagen I fiber bundles; dc, dense collagen I fiber bundles; el, elastin layer; fb, fibroblasts; nc, notochord cells; ne, notochord epithelium; ns, notochord sheath; Ob, osteoblasts; ot, osteocytes; 4PBA, 4-phenylbutyrate.

The effect of the treatments on vertebral thickness was also addressed by a deeper 2D analysis along the whole vertebral length. The values were compared following the arbitrary division of the vertebra in 3 regions—namely, aEP, centrum, and cEP. A reduced thickness was detected in the selected vertebral regions of both mutants compared with WT (Figure 2A i, B i; Figure S6A, B). Confirming 3D μCT data, only ALN significantly increased the vertebral thickness in *Chi/+* zebrafish compared with controls and this effect was driven by the improvement in cEP endplates (Figure 2A ii, Table S3A, Figure S6A). No changes in vertebral thickness were observed in *p3h1^−/−^*-treated fish (Figure 2B ii, Table S3B, Figure S6B).

Intervertebral disc morphology was then investigated by histological staining. The IVD is an organized structure that connects consecutive vertebrae endplates and acts as shock-absorbing cushions to limit mechanical stress^22^ and its degeneration is a common consequence of the long-term effects of poor bone quality.^23^ Wild-type IVD is composed of collagen II embedded in a matrix resembling cartilage, elastin, and collagen I fiber bundles, from inside to outside.^24^ Intervertebral disc enlargement and dislocation were only detectable in dominant *Chi/+* zebrafish, but not in *p3h1^−/−^*(Figure 2C, D i; Figure S7 i, ii). Alendronate and 4PBA + ALN significantly ameliorated IVD morphology with respect to untreated controls (Figure 2C, D ii).

To assess the effects of the treatments on vertebral morphology, we analyzed the shape of the vertebral autocentrum, the central body of the vertebra. This was done using landmark-based geometric morphometrics, a technique that quantifies shape variations by placing specific reference points (landmarks) on images of the structure of interest. In this study, landmarks were positioned on 2D μCT images of reconstructed vertebrae. The analysis revealed a clear difference in landmark distribution between *Chi/+* and WT, as shown in the scatterplots (Figure 2E i). In *Chi/+* fish, the distribution of landmarks was more variable compared with WT, indicating an altered vertebral shape. In contrast, *p3h1^−/−^* mutants showed only mild shape variability, as reflected in the confidence ellipses (Figure 2F i).

Interestingly, all treatments reduced the dispersion of landmarks in *Chi/+* fish, suggesting a partial normalization of vertebral shape compared with untreated siblings (Figure 2E ii). In contrast, no appreciable effect on dispersion was observed in *p3h1^−/−^* mutants (Figure 2F ii).

The 2D findings supported a major role of ALN in the vertebral thickening, although in specific bone regions in *Chi/+* fish, and a positive effect of ALN and 4PBA + ALN in rescue IVD morphology in *Chi/+* fish*.* Furthermore, a qualitative evaluation of the vertebral shape supports a positive effect of the 3 treatments in the dominant model.

### 4PBA and/or ALN show opposite effects on osteoclast activity in dominant and recessive OI zebrafish models

**Figure 3:**
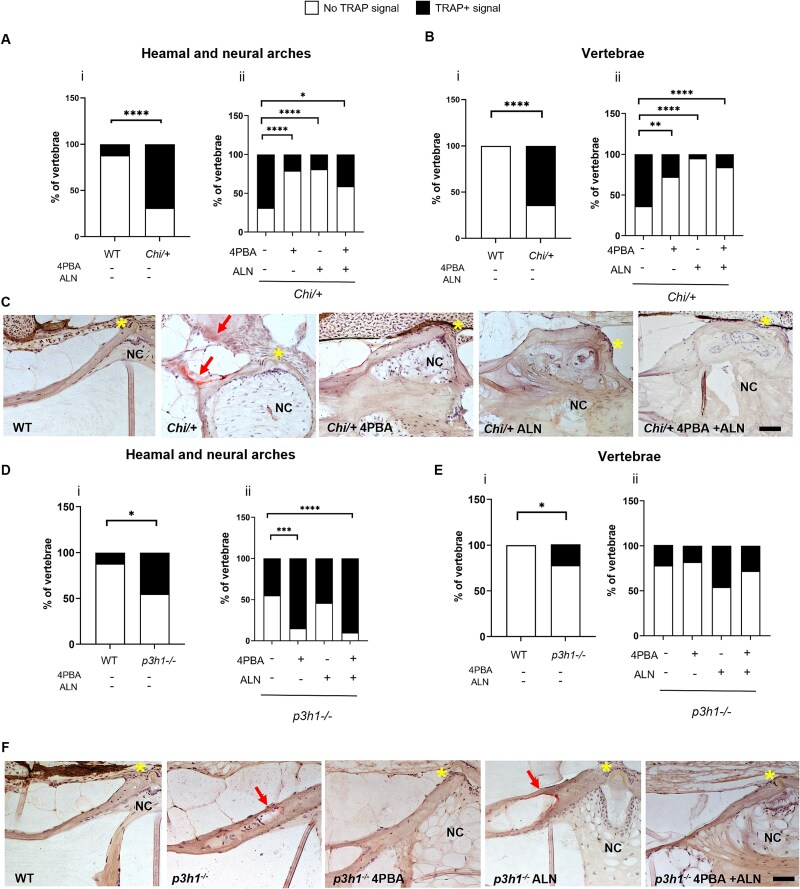
Analysis of osteoclast activity in *Chi/+* and *p3h1^−/−^* zebrafish. (A, B) TRAP staining quantification on haemal and neural arches and autocentrum of WT and *Chi/+* (i) and *Chi/+* untreated and treated with 4PBA, ALN, and 4PBA + ALN (ii). (C) Representative images of TRAP-stained WT and *Chi/+* caudal vertebrae. (D, E) TRAP staining quantification on arches and vertebrae of WT and *p3h1^−/−^* (i) and *p3h1^−/−^* untreated and treated with 4PBA, ALN, and 4PBA + ALN (ii). (F) Representative images of TRAP-stained WT and *p3h1^−/−^* caudal vertebrae. Red arrows indicate TRAP signal on vertebral bodies. The yellow asterisks indicate the intervertebral disc. Scale bar: 50 μm. In the graphs, the percentage of TRAP+ vertebrae on total number of analyzed vertebrae is reported. ^*^p < .05, ^**^p < .01, ^***^p < .001, ^****^p < .0001. Abbreviations: ALN, alendronate; NC, notochord; TRAP, tartrate-resistant acid phosphatase; 4PBA, 4-phenylbutyrate.

To investigate the effect of 4PBA-only, ALN-only, and combined treatment on OC homeostasis, TRAP activity was evaluated on histological sections. TRAP was detectable in the haemal and neural arches, where OC differentiation initially occurs during zebrafish development, as well as in the trabeculae and autocentrum. In *Chi/+* fish, an increased TRAP signal was observed in both sites compared with WT, but it significantly decreased across all treatment groups (Figure 3A-C). In *p3h1^−/−^* the signal was also higher in both arches and vertebral body compared with WT, revealing for the first time the presence of increased TRAP enzymatic activity in the recessive OI type VIII zebrafish model (Figure 3D i, E i, F). Unexpectedly, 4PBA-only and 4PBA + ALN caused a significant increase in the signal in the arches compared with untreated mutants (Figure 3D ii, E ii, F).

Gene expression of OC markers was also evaluated. In *Chi/+* only, the *rankl/opg* ratio increased with respect to WT, in line with histological observation, whereas **a*cp5a* expression was reduced in *p3h1^−/−^*. The effects of treatments were limited to 4PBA that stimulated **o*pg* expression in *Chi/+* and 4PBA + ALN that increased *rankl/opg* ratio in *p3h1^−/−^* (Figure S8, Figure S9).

Altogether, these data supported an increase TRAP enzymatic activity in both dominant and recessive OI mutants. Surprisingly, an opposite response to the treatments was detected. In *Chi/+* fish all of the drugs reduced TRAP activity, whereas in *p3h1^−/−^* fish, 4PBA-only and 4PBA + ALN increased the signal in the arches.

### The combination of 4PBA and ALN positively affects osteoblast differentiation, activity, and ER stress in both dominant and recessive OI

To dissect the effect of the treatments on osteoblast differentiation and activity, the expression of the osteoblast markers **b*glap* (osteocalcin) and **c*ol1a1a* was evaluated in WT, *Chi/+* and *p3h1^−/−^*.

**Figure 4:**
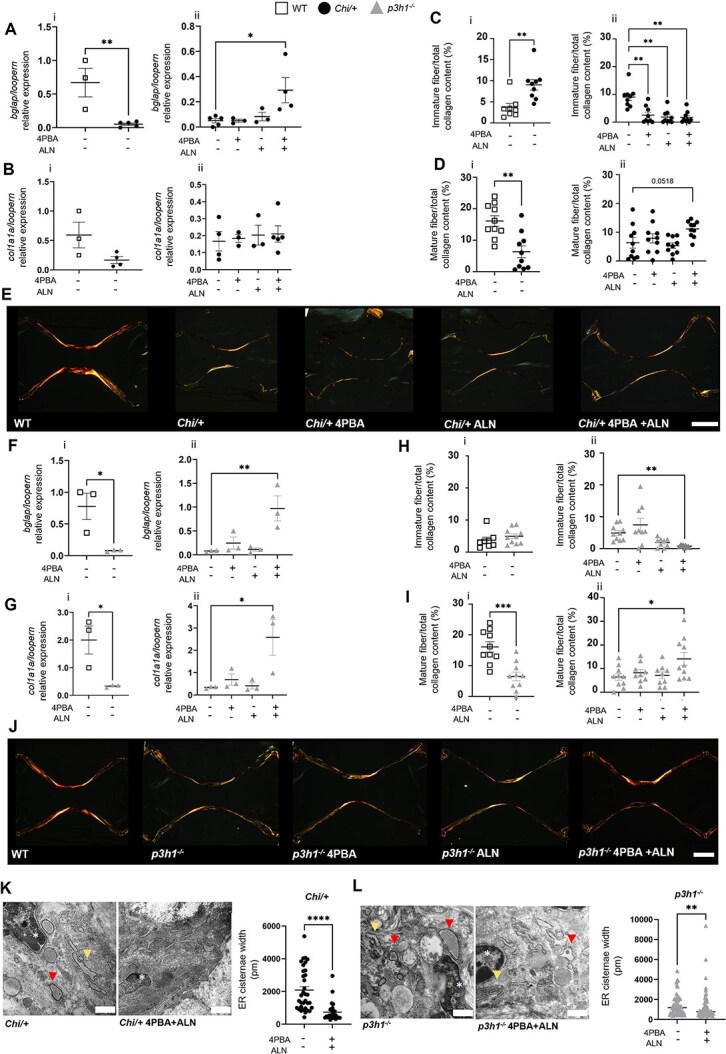
Analysis of osteoblast homeostasis and extracellular collagen maturation. (A) RT-qPCR analysis of **b*glap* expression in WT, *Chi/+* (i), and untreated and treated *Chi/+* (ii). (B) RT-qPCR analysis of **c*ol1a1a* expression in WT, *Chi/+*, and untreated and treated *Chi/+*. (C, D) Quantification of the immature (green-yellow) or mature (red) collagen fibers on total collagen content in WT, *Chi/+* (i) and untreated and treated *Chi/+* (ii) caudal vertebrae. (E) Representative images of picrosirius red–stained caudal vertebrae sections of WT and untreated or treated *Chi/+*. Scale bar: 100 μm. (F) RT-qPCR analysis of **b*glap* expression in WT, *p3h1^−/−^* (i), and untreated and treated *p3h1^−/−^* (ii). (G) RT-qPCR analysis of **c*ol1a1a* expression in WT, *p3h1^−/−^* (i), and untreated and treated *p3h1^−/−^* (ii). (H, I) Quantification of the immature (green-yellow) or mature (red) collagen fibers on total collagen content in WT, *p3h1^−/−^* (i), and untreated and treated *p3h1^−/−^* caudal vertebrae (ii). (J) Representative images of picrosirius red–stained caudal vertebrae sections of WT and untreated and treated *p3h1^−/−^*. Scale bar: 100 μm. (K) Transmission electron microscopy representative images of *Chi/+* osteoblasts and quantification of ER cisternae width in *Chi/+* and *Chi/+* treated with 4PBA + ALN. Scale bar = 500 nm. (L) Transmission electron microscopy representative images of *p3h1^−/−^* osteoblasts and quantification of ER cisternae width in *p3h1^−/−^* and *p3h1^−/−^* treated with 4PBA + ALN. Scale bar = 500 nm. (Red arrowheads indicate ER cisternae transverse sections, yellow arrowheads indicate ER cisternae sagittal sections, pink asterisks indicate osteoblasts nuclei.). ^*^p < .05, ^**^p < .01, ^***^p < .001, ^****^p < .0001. Abbreviations: ALN, alendronate; ER, endoplasmic reticulum; RT-qPCR, reverse transcriptase quantitative PCR; 4PBA, 4-phenylbutyrate.

Both *Chi/+* and *p3h1^−/−^* showed a strong downregulation of **b*glap* compared with WT, supporting a delay in osteoblast maturation in the OI models (Figure 4A i, F i). Only 4PBA + ALN increased **b*glap* expression level in both mutants (Figure 4A ii, F ii). Also, a reduced expression of **c*ol1a1a* was observed in the *p3h1^−/−^* model compared with WT (Figure 4B i, G i). The treatments did not ameliorate collagen I expression in *Chi/+*, while a significant upregulation was detected in *p3h1^−/−^* after 4PBA + ALN administration (Figure 4B ii, G ii).

Previous in vitro and in vivo studies showed a positive effect of 4PBA on collagen I secretion and deposition in the extracellular matrix.^3^^,^^7^^,^^12^^,^^13^ Thus, the maturation of collagen I fibers was evaluated in caudal vertebral matrix by picrosirius red staining. A significant increase in immature and a decrease in mature collagen fiber area were detected in *Chi*/+ compared with WT zebrafish (Figure 4C i, D i, E), while only a significant reduction in mature fiber area was detectable in *p3h1^−/−^* zebrafish compared with WT (Figure 4H i, I i, J). In *Chi/+* fish, all treatments significantly reduced the immature fiber area compared with untreated mutant fish, while only the combined treatment slightly improved the mature fiber area (*p* = .0518) (Figure 4C ii, D ii, E). In *p3h1^−/−^* fish, only the combined treatment was effective in reducing immature and increasing the mature fiber area (Figure 4H ii, I ii, J).

Endoplasmic reticulum stress associated with enlarged ER cisternae was previously described in *Chi/+* and *p3h1^−/−^* zebrafish.^7^^,^^9^^,^^19^ To evaluate the effect of the treatment on osteoblast homeostasis, ER cisternae size was analyzed using transmission electron microscopy (TEM) images of 4PBA + ALN–treated and untreated mutants. Interestingly, a significant reduction in ER cisternae size was observed in both OI models (Figure 4K, L).

The data support the value of 4PBA + ALN treatment in reducing ER stress by improving osteoblast differentiation and activity and promoting collagen secretion and maturation in the extracellular matrix.

### 4PBA ameliorates osteocyte viability in both dominant and recessive OI zebrafish models

**Figure 5:**
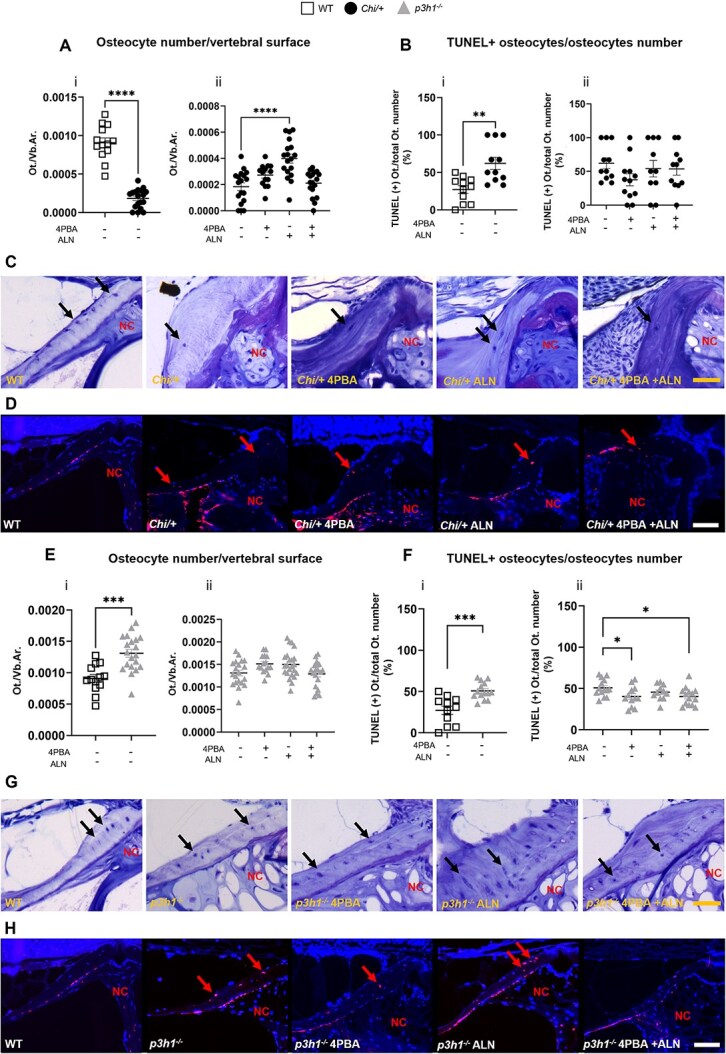
Analysis of osteocyte number and viability in *Chi/+* and *p3h1^−/−^*. (A) Osteocyte number normalized to vertebral area (Ot./vb.Ar.) performed on toluidine blue sections of WT, *Chi/+* (i), and untreated and treated *Chi/+* (ii). (B) TUNEL+ osteocytes on total osteocytes number (TUNEL(+) Ot./ total Ot. number) in WT, *Chi/+* (i), and untreated and treated *Chi/+* (ii). (C) Representative images of toluidine blue–stained vertebrae showing osteocytes (black arrows) in WT and untreated or treated *Chi/+.* Scale bar: 50 μm. (D) Representative images of TUNEL assay performed on WT and treated and untreated *Chi/+* vertebrae. Red arrows indicate apoptotic osteocytes; nuclei are stained with DAPI (4′,6-diamidino-2-phenylindole). Scale bar: 100 μm. (E) Osteocyte number on vertebral area (Ot./vb.Ar.) performed on toluidine blue–stained sections of WT, *p3h1−/−* (i), and untreated and treated *p3h1^−/^* (ii). (F) TUNEL+ osteocytes on total osteocyte number (TUNEL(+) Ot./ total Ot. Number) in WT *p3h1^−/−^* (i) and untreated and treated *p3h1^−/−^* (ii). (G) Representative images of toluidine blue–stained vertebrae showing osteocytes (black arrows) in WT and untreated or treated *p3h1^−/−^*. Scale bar: 50 μm. (H) Representative images of TUNEL assay performed on WT and treated and untreated *p3h1^−/−^* vertebrae. Red arrows indicate apoptotic osteocytes; nuclei are stained with DAPI. Scale bar: 100 μm. In the graphs, each dot represents a single value. ^*^p < .05, ^**^p < .01, ^***^p < .001, ^****^p < .0001. Abbreviations: ALN, alendronate; NC, notochord; 4PBA, 4-phenylbutyrate.

Histological analysis of toluidine blue–stained WT and mutant vertebral sections was performed to evaluate the number of osteocytes (Ot) per area of vertebral body (N.Ot/Vb.Ar). In *Chi/+* vertebrae, osteocyte number was reduced compared with WT (Figure 5A i, C) and only ALN alone administration increases this value (Figure 5A ii, C). On the contrary, a significant increased number of osteocytes was detected in *p3h1^−/−^* vertebrae compared with WT (Figure 5E i, G). None of the treatments affected this value (Figure 5E ii, G).

A TUNEL assay was performed to investigate osteocyte viability in both *Chi/+* and *p3h1^−/−^* treated and untreated fish. An increased percentage of apoptotic osteocytes was detected in both *Chi/+* (Figure 5B i, D) and *p3h1^−/−^* vertebrae (Figure 5F i, H) compared with WT. Interestingly, only 4PBA alone or in combination with ALN significantly reduced the number of apoptotic cells in *p3h1^−/−^* vertebrae (Figure 5B ii, F ii, D, H).

These data revealed an abnormal and opposite osteocyte number in *Chi/+* and *p3h1^−/−^* zebrafish models compared with WT, with a decrease in the former and an increase in the latter. Nevertheless, in both models, an increased osteocyte apoptosis activation was evident. Interestingly, 4PBA and 4PBA + ALN increased the osteocyte viability in the *p3h1^−/−^* model.

## Discussion

In this study, we utilized the well-characterized OI zebrafish models *Chihuahua* (*Chi/+*) as a dominant form and *p3h1^−/−^* as a recessive form, to investigate the tissue and cellular effects of ALN and 4PBA administered individually or in combination. The enhanced mineralization observed with the combined treatment, compared with single-drug administration in *Chi/+* larvae cranial bones, prompted further research in adult fish. The 2-mo treatment in adult fish primarily influenced the cellular components and the organic extracellular matrix. In OI, the imbalance between bone formation and bone resorption occurs when altered matrix deposition, as consequence of an impairment of osteoblast differentiation and functionality, is coupled with an increased osteoclast number and/or activity.^25^ The downregulation of the expression of osteocalcin (**b*glap*), an osteoblast differentiation and maturation marker, already described in *Chi/+* dermal fin rays^14^ was here further confirmed. Furthermore, a significant reduced expression of **b*glap* was for the first time also shown in the recessive *p3h1^−/−^* fish. Only 4PBA + ALN administration rescued **b*glap* expression in both models.

The effect of ALN on inducing osteogenic differentiation has been reported both in human bone marrow stromal cells and in biomineralized microspheres.^26^^,^^27^ Also, ALN partially restored osteoblast function in an osteoporosis-induced zebrafish model.^28^ Similarly, the ability of 4PBA in favoring osteoblast differentiation and activity has been described in OI models both in vitro and in vivo.^3^^,^^7^^,^^12^^,^^13^ Of relevance, our results provide the first clear evidence of the synergistic effect of the combined treatment with ALN and 4PBA at the osteoblast level.

In both zebrafish OI models, only the combined administration of 4PBA and ALN led to the formation of thicker, more mature collagen fibers and a reduction in ER cisternae size. This suggests that the proteostasis-regulating activity of 4PBA facilitated the secretion of collagen molecules capable of properly assembling into fibrils, thereby enhancing the ALN effect in reducing bone turnover.^13^

In OI, the decrease in mineral apposition rate in the presence of increased number of bone remodeling units points to an imbalance in bone remodeling in favor of osteoclast activity.^29^ In the *Brtl* OI mouse model, osteoblast surface/bone surface is similar to WT or slightly decreased after puberty, in the presence of an increased number of active osteoclasts.^30^ Elevated levels of serum TRAP were observed in OI type VIII patients with null mutations in *P3H1* despite a normal osteoclast count.^31^ Bone turnover was normal in *P3H1* KO mice, while bone formation was reduced, and osteoblast and osteoclast indices were reported as normal to low.^32^

TRAP staining performed on both *Chi/+ and p3h1^−/−^* vertebral sections showed increased osteoclast activity in both dominant and recessive OI models compared with WT. The gene expression data did not align with the histological findings, but this is not uncommon. Often, transcriptomic data do not correlate directly with proteomic data or enzymatic activity.

Based on the inhibitory action of ALN on osteoclasts and the previously reported inhibition of bone resorption by 4PBA, demonstrated both in vitro and in vivo,^7^^,^^12^^,^^13^ all of the treatments significantly reduced the high osteoclast activity in *Chi/+*, as expected. Nevertheless, unexpectedly, in *p3h1^−/−^* fish, ALN did not modulate TRAP signals and 4PBA and 4PBA + ALN increased osteoclast activity. In addition to mammalian multinucleated osteoclasts, at the level of the arches zebrafish also have mononucleated osteoclasts, characterized by low bone resorption activity and mainly involved in bone growth during early skeletal development.^33^ Although several biochemical and functional features are shared between typical mammalian osteoclasts and mononucleated osteoclasts, the existence of morphological dissimilarities must be considered. Furthermore, it is known that osteocytes can actively remove their perilacunar matrix and minerals via acidification and TRAP-mediated proteolytic degradation^34^ in a process called osteocytic osteolysis. Since our data revealed, for the first time, an increased number of osteocytes in *p3h1^−/−^* vertebral bodies, we can speculate that their resorptive activity could be enhanced as a compensatory reaction to osteoclast inhibition caused by ALN.

Osteocyte number, size, and function are affected in OI, as reported for OI type I and V, which are characterized by an increased osteocyte lacunar density.^35^^,^^36^ The *oim/oim* bone has more osteocyte lacunae and more numerous and branched vascular canals compared with WT.^37^ As previously observed,^6^ the number of osteocytes is severely reduced in *Chihuahua* vertebrae compared with WT. On the other hand, we revealed for the first time an increased number of osteocyte/vertebra area in *p3h1^−/−^*, in line with the increased osteocyte lacunar density found in several OI types.^35^^,^^36^^,^^38^ Interestingly, apoptosis of *Chi/+* and *p3h1^−/−^* osteocytes was significantly increased compared with WT and only 4PBA and 4PBA + ALN were able to reduce cellular death only in the recessive OI form. We cannot exclude that apoptosis activation in *p3h1^−/−^* zebrafish could be linked to the impairment of other proteins requiring proline 3 hydroxylation. For instance, in the absence of prolyl 3-hydroxylase, Hypoxia-Inducible Factor 1-alpha (HIF-1α) may become, even if indirectly, stabilized due to the impaired hydroxylation process, leading to its accumulation even under normoxic conditions. This persistent stabilization can activate apoptotic pathways, as the prolonged activation of HIF-1α may induce cellular stress responses, including the disruption of metabolic balance and the accumulation of reactive oxygen species, which, in turn, can trigger programmed cell death.^39^

The effects of the treatments at the tissue level were mildly limited compared with expectations. The reduced bone volume and BMD observed in both models via μCT were not improved by any of the treatments, and a significant increase in vertebral thickness was only evident following ALN administration.

The combined administration of BPs and anabolic agents has been previously explored, specifically through the use of zoledronate and sclerostin antibody (Scl-Ab), which led to improvements in tibial bone geometrical parameters in an Amish mouse model of OI.^40^ In addition, synergistic effects of Scl-Ab combined with low doses of pamidronate improved bone mass and trabecular thickness in the *Brtl* mouse, suggesting that the modulation of BP concentration allows the anabolic drug’s effect to emerge in the combined treatment.^41^

The absence of positive results regarding bone geometrical properties in our study may be attributed to the age of the fish and/or the duration of the treatment. Due to technical limitations, we were only able to inject 6-mo-old fish, and their growth during the 2-mo treatment period may not have been sufficient to elicit a significant effect on bone properties. Nevertheless, at least in the more severely affected *Chi/+* model, an amelioration of vertebral shape following all treatments was suggested, as indicated by reduced dispersion of the landmarks. Nevertheless, no effect on bone fracture was evident (data not shown).

Lumbar spine vertebral bodies of children and adolescents with moderate to severe OI are characterized by an abnormal shape, worsening over time, and one of the treatment outcomes with BPs is the reduction in vertebral compressions and the decrease in scoliosis.^1^ Abnormal vertebral shape was clearly observed in *Chi/+* compared with WT models, and likely the increased bone thickness induced by antiresorptive activity of ALN positively impacts on this condition, contributing also to the amelioration of intervertebral disc conformation.

In conclusion, we presented a comprehensive analysis of bone quality and cellular homeostasis in two zebrafish OI models with distinct genetic defects, aiming to assess the combined effects of ALN and 4PBA on the skeleton. We demonstrated a synergistic effect of the drugs in enhancing osteoblast differentiation and collagen secretion and maturation, whereas mutation-dependent effects were detected on osteoclast and osteocyte parameters. At the tissue level only the 2-mo ALN treatment resulted in an improvement in bone thickness. Nevertheless, all treatment regimens suggested an amelioration in vertebral shape only in the more severe dominant OI model. The differences observed between the two models, coupled with the variability in treatment responses, underscore the importance of a thorough understanding of model-specific OI pathophysiology and the need to define the optimal treatment duration to develop effective therapeutic strategies.

## Supplementary Material

Masiero_et_al_supplementary_Material_JBMRPlus_revision_2_ziaf112

## Data Availability

All relevant data are present in the manuscript and in the supplementary material; raw data are available upon request from the corresponding authors.
