# Supplementary material for: Combined antiresorptive and new anabolic drug approach in osteogenesis imperfecta zebrafish models
Source: JBMR Plus. 2025 Jul 2;9(9):ziaf112. doi: 10.1093/jbmrpl/ziaf112 (PMC12356371; doi:10.1093/jbmrpl/ziaf112)
Supplement: Masiero_et_al_supplementary_Material_JBMRPlus_revision_2_ziaf112 [file masiero_et_al_supplementary_material_jbmrplus_revision_2_ziaf112.pdf]

Supplementary material

A

Genotype	Treatment	Age	Tissues	Experiment	Fish number	Vertebrae/IVD number (total)	Slide number per vertebra
WT	Placebo	7 dpf	Whole forming-embryo	Toxicity test	10	/	/
		8 mpf	Caudal vertebrae	μCT	6-7	8	/
				Geometric morphometrics	5	50	/
				Toluidine blue (IVD analysis)	3	66 IVDs	1 (midline sagittal plane)
				Toluidine blue (osteocyte number analysis)	3	13	2 (midline sagittal plane)
				TRAP	3	24	5-7
				Picrosirius red staining	3	9	1 (midline sagittal plane)
				TUNEL	3	11	2 (consecutive slides of midline sagittal plane)
			Skull and precaudal vertebrae	RT-qPCR	3-4 (3 technical replicates for each sample)	/	/
	4PBA	8 mpf	Caudal vertebrae	μCT	7	8	/
	ALN	7 dpf	Whole forming-embryo	Toxicity test	30 (10 for each tested dose)	/	/
		8 mpf	Caudal vertebrae	μCT	7	8	/
	4PBA + ALN	8 mpf	Caudal vertebrae	μCT	10	11	/

B

Genotype	Treatment	Age	Tissues	Experiment	Fish number	Vertebrae/IVD number (total)	Slide number per vertebra
Chi/+	Placebo	11 dpf	Whole larvae	ARS	52	/	/
		8 mpf	Caudal vertebrae	μCT	8	8	/
				Geometric morphometrics	5	50	/
				Toluidine blue (IVD analysis)	3	82 IVDs	1 (midline sagittal plane)
				Toluidine blue (osteocyte number analysis)	4	18	2 (midline sagittal plane)
				TRAP	3	36	5-7
				Picrosirius red staining	3	9	1 (midline sagittal plane)
				TUNEL	3	11	2 (consecutive slides of midline sagittal plane)
				TEM	1	1 (27 cisternae)	1
			Skull and precaudal vertebrae	RT-qPCR	3-4 (3 technical replicates for each sample)	/	/
	4PBA	11 dpf	Whole larvae	ARS	48	/	/
		8 mpf	Caudal vertebrae	μCT	6-7	7	
				Geometric morphometrics	6	50	/

				Toluidine blue (IVD analysis)	4	82 IVDs	1 (midline sagittal plane)
				Toluidine blue (osteocyte number analysis)	3	14	2 (midline sagittal plane)
				TRAP	3	43	5-7
				Picrosirius red staining	4	9	1 (midline sagittal plane)
				TUNEL	3	12	2 (consecutive slides of midline sagittal plane)
			Skull and precaudal vertebrae	RT-qPCR	3-4 (3 technical replicates for each sample)	/	/
	ALN	11 dpf	Whole larvae	ARS	47	/	/
		8 mpf	Caudal vertebrae	μCT	6	6	/
				Geometric morphometrics	5	50	/
				Toluidine blue (IVD analysis)	4	82 IVDs	1 (midline sagittal plane)
				Toluidine blue (osteocyte number analysis)	3	18	2 (midline sagittal plane)
				TRAP	3	43	5-7
				Picrosirius red staining	3	9	1 (midline sagittal plane)
				TUNEL	3	11	2 (consecutive slides of midline sagittal plane)
			Skull and precaudal vertebrae	RT-qPCR	3-4 (3 technical replicates for each sample)	/	/
	4PBA +ALN	11 dpf	Whole larvae	ARS	53	/	/
		8 mpf	Caudal vertebrae	μCT	8-9	9	/
				Geometric morphometrics	5	50	/
				Toluidine blue (IVD analysis)	4	82 IVDs	1 (midline sagittal plane)
				Toluidine blue (osteocyte number analysis)	3	18	2 (midline sagittal plane)
				TRAP	3	37	5-7
				Picrosirius red staining	3	9	1 (midline sagittal plane)
				TUNEL	3	11	2 (consecutive slides of midline sagittal plane)
				TEM	1	1 (34 cisternae)	1
			Skull and precaudal vertebrae	RT-qPCR	3-4 (3 technical replicates for each sample)	/	/

C

Genotype	Treatment	Age	Tissues	Experiment	Fish number	Vertebrae/IVD number (total)	Slide number per vertebra
p3h1$^{-/-}$	Placebo	8 mpf	Caudal vertebrae	μCT	5	5	/
				Geometric morphometrics	5	50	/
				Toluidine blue (IVD analysis)	3	70 IVDs	1 (midline sagittal plane)
				Toluidine blue (osteocyte number analysis)	3	20	2 (midline sagittal plane)
				TRAP	3	40	5-7
				Picrosirius red staining	3	10	1 (midline sagittal plane)
				TUNEL	3	14	2 (consecutive slides of midline sagittal plane)
				TEM	1	1 (69 cisternae)	1
			Skull and precaudal vertebrae	RT-qPCR	3-4 (3 technical replicates for each sample)	/	/
	4PBA	8 mpf	Caudal vertebrae	μCT	7	7	/
				Geometric morphometrics	5	50	/
				Toluidine blue (IVD analysis)	3	76 IVDs	1 (midline sagittal plane)
				Toluidine blue (osteocyte number analysis)	3	15	2 (midline sagittal plane)
				TRAP	3	39	5-7
				Picrosirius red staining	3	9	1 (midline sagittal plane)
				TUNEL	3	12	2 (consecutive slides of midline sagittal plane)
			Skull and precaudal vertebrae	RT-qPCR	3-4 (3 technical replicates for each sample)	/	/
	ALN	8 mpf	Caudal vertebrae	μCT	6-7	7	/
				Geometric morphometrics	5	50	/
				Toluidine blue (IVD analysis)	3	50 IVDs	1 (midline sagittal plane)
				Toluidine blue (osteocyte number analysis)	3	23	2 (midline sagittal plane)
				TRAP	3	35	5-7
				Picrosirius red staining	3	9	1 (midline sagittal plane)
				TUNEL	3	11	2 (consecutive slides of midline sagittal plane)
			Skull and precaudal vertebrae	RT-qPCR	3-4 (3 technical replicates for each sample)	/	/
	4PBA +ALN	8 mpf	Caudal vertebrae	μCT	8	8	/
				Geometric morphometrics	5	50	/
				Toluidine blue (IVD analysis)	3	78 IVDs	1 (midline sagittal plane)
				Toluidine blue (osteocyte number analysis)	3	19	2 (midline sagittal plane)
				TRAP	3	39	5-7
				Picrosirius red staining	3	10	1 (midline sagittal plane)
				TUNEL	3	12	2 (consecutive slides of midline sagittal plane)
				TEM	1	1 (77 cisternae)	1

			Skull and precaudal vertebrae	RT-qPCR	3-4 (3 technical replicates for each sample)	/	/

Supplementary Table 1. Details of animal groups used in the study. (**A**) WT, (**B**) *Chi/+* and (**C**) *p3h1*$^{-/-}$ fish used in the study. 4PBA = 4-phenylbutyrate; ALN = alendronate; dpf = days post fertilization; mpf = months post fertilization; μCT = micro-computed tomography; RT-qPCR = quantitative real-time polymerase chain reaction; TEM = transmission electron microscopy.

Gene	Ensembl ID	Annealing temperature	Sequence (5'→ 3')	Ref
bglap	ENSDARG00000058414	60 °C	FW: CCTGATGACTGTGTGTCTGAG;<br>RV: CGCTTCACAAACACACCTTC	1
col1a1a	ENSDARG00000012405	60 °C	FW: CTGCAAGAACAGCATTGCAT<br>RV: TAGGCAGACGGGATGTTTTC	2
acp5a	ENSDARG00000019763	60 °C	FW: GCCAAAACTGCTTCTCAGATGG;<br>RV: GACGTCTTCAAAGGTTTCCTGG	4
ctsk	/ ENSDART00000011497.8	60 °C	FW: GGATCAGCAGTGTGCCTACA;<br>RV: TGGACTGCATGGCATCTATG	/
loopern4 S	expressed repeat element (not in the database)	60 °C	FW: TGAGCTGAAACTTTACAGACACAT;<br>RV: AGACTTTGGTGTCTCCAGAATG	3
opg	ENSDARG00000098377	60 °C	FW: GGGTCATTTCTCAGACGCCA;<br>RV: CGGGTCGAATACAGCAGTCC	4
rankl	ENSDARG00000068141	60 °C	FW: CTCACCTTCCAATCAAGACGCCC;<br>RV: CTTTCATGCCATCCCAGGCTATCT	4

Supplementary Table 2. List of primers used for RT-qPCR. The reference is indicated for primers taken from the literature.

A

	**Abdominal endplate**	**Vertebral centrum**	**Caudal endplate**
**WT vs *Chi/+* (mm)**	0.030 ± 0.002<br>vs<br>0.024 ± 0.001<br>***	0.025 ± 0.002<br>vs<br>0.018± 0.002<br>****	0.032 ± 0.002<br>vs<br>0.028 ± 0.004
***Chi/+* vs *Chi/+* ALN (mm)**	0.024 ± 0.001<br>vs<br>0.028 ± 0.004	0.018± 0.002<br>vs<br>0.018 ± 0.001	0.028 ± 0.004<br>vs<br>0.034 ± 0.006<br>*
***Chi/+* vs *Chi/+* 4PBA (mm)**	0.024 ± 0.001<br>vs<br>0.025± 0.004	0.018± 0.002<br>vs<br>0.016 ± 0.001	0.028 ± 0.004<br>vs<br>0.029 ± 0.007
***Chi/+* vs *Chi/+* 4PBA+ALN (mm)**	0.024 ± 0.001<br>vs<br>0.028±0.004	0.018± 0.002<br>vs<br>0.018 ± 0.001	27,72 ± 6,16<br>vs<br>0.029±0.006

B

	**Abdominal endplate**	**Vertebral centrum**	**Caudal endplate**
**WT vs $p3h1^{-/-}$ (mm)**	0.031 ± 0.001<br>vs<br>0.029 ± 0.003	0.028 ± 0.001<br>vs<br>0.025 ± 0.002<br>*	0.033 ± 0.001<br>vs<br>0.029± 0.002<br>**
**$p3h1^{-/-}$ vs $p3h1^{-/-}$ ALN (mm)**	0.029 ± 0.003<br>vs<br>0.029 ± 0.002	0.028 ± 0.001<br>vs<br>0.026±0.001	0.033 ± 0.001<br>vs<br>0.030± 0.002
**$p3h1^{-/-}$ vs $p3h1^{-/-}$ 4PBA (mm)**	0.029 ± 0.003<br>vs<br>0.029 ± 0.004	0.028 ± 0.001<br>vs<br>0.024± 0001	0.033 ± 0.001<br>vs<br>0.029 ± 0.002
**$p3h1^{-/-}$ vs $p3h1^{-/-}$ 4PBA+ALN (mm)**	0.029 ± 0.003<br>vs<br>0.028± 0.002	0.028 ± 0.001<br>vs<br>0.025 ± 0.002	0.033 ± 0.001<br>vs<br>0.030 ± 0.002

Supplementary Table 3. Statistical analysis of 2D thickness distribution in vertebral centrum and endplates. (**A**) Comparison between untreated WT and *Chi/+* and untreated and treated *Chi/+*. (**B**) Comparison between untreated WT and $p3h1^{-/-}$ and untreated and treated $p3h1^{-/-}$. * P < 0.05, ** P < 0.01,*** P < 0.001, **** P < 0.0001.

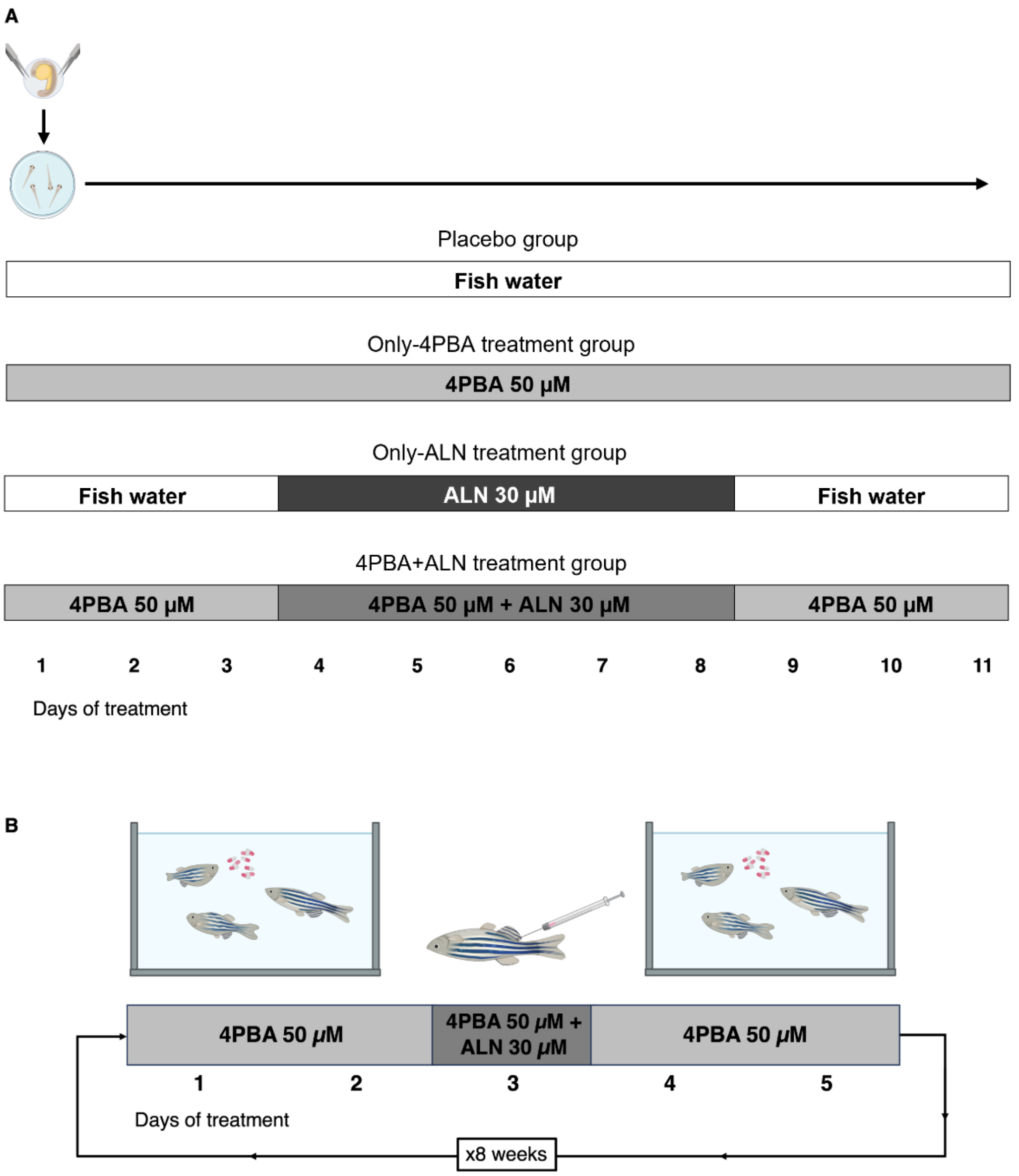

Supplementary Figure 1. Combined drug administration schemes. (**A**) *Chi/+* embryos were treated from 1 to 3 dpf with 50 µM 4PBA, from 4 to 8 dpf with 50 µM 4PBA and 30 µM ALN, and from 9 to 11 dpf with 50 µM 4PBA. At 11 dpf fish were euthanized and fixed in 4% PFA in PBS. The drugs were dissolved in fish water (**B**) WT, *Chi/+* and *p3h1*$^{-/-}$ fish were treated from 6 to 8 mpf. 50 µM 4PBA was dissolved in fish water and half of the solution was changed every day for 5 days/week. 5µL of 30 µM ALN solution was intraperitoneally injected once a week for 8 weeks. At 8 mpf fish were euthanized and processed accordingly to established protocols. Treatment groups were placebo, 4PBA-only, ALN-only and 4PBA+ALN treated fish per each genotype. *Created with biorender.com*

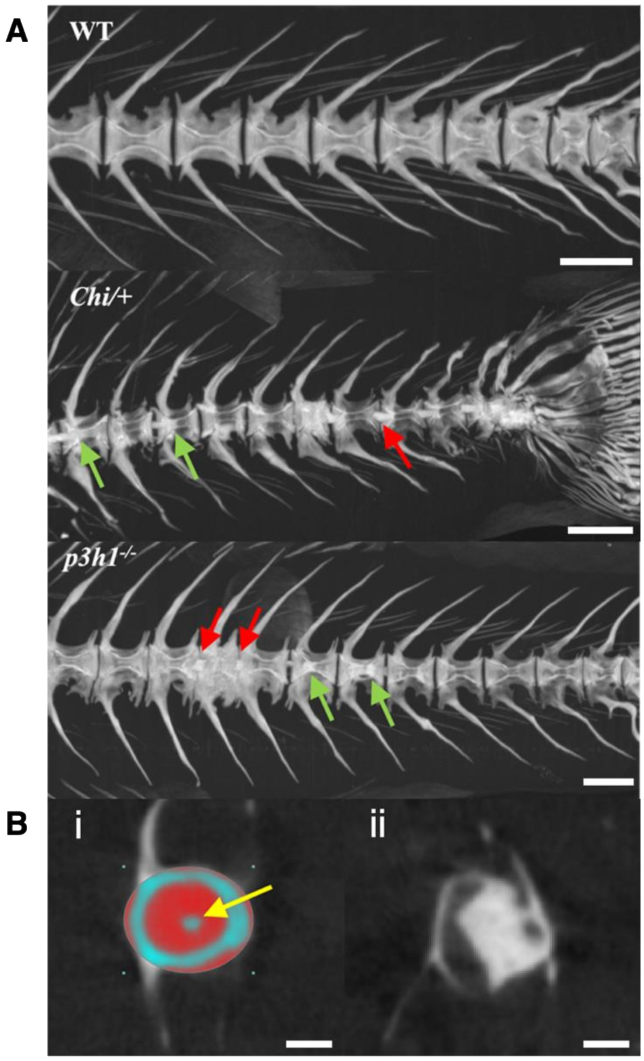

Supplementary Figure 2. Region of interest selection on µCT reconstructed vertebrae. (**A**) µCT reconstructed caudal vertebrae of WT, *Chi/+* and *p3h1*$^{-/-}$ showing ectopically accumulated mineralized tissue located in the intervertebral space (red arrows) and inside the vertebral centra (green arrows) of mutant fish. Scale bar: 500 µm. (**B**) Axial view of reconstructed caudal vertebrae. (**i**) The region of interest (ROI, red area) was selected and mineralized mass (yellow arrow) was subsequently manually excluded. (**ii**) When the lumps of mineralization were not discernible from the vertebral centrum, vertebrae were excluded from the analysis. Scale bar: 70 µm.

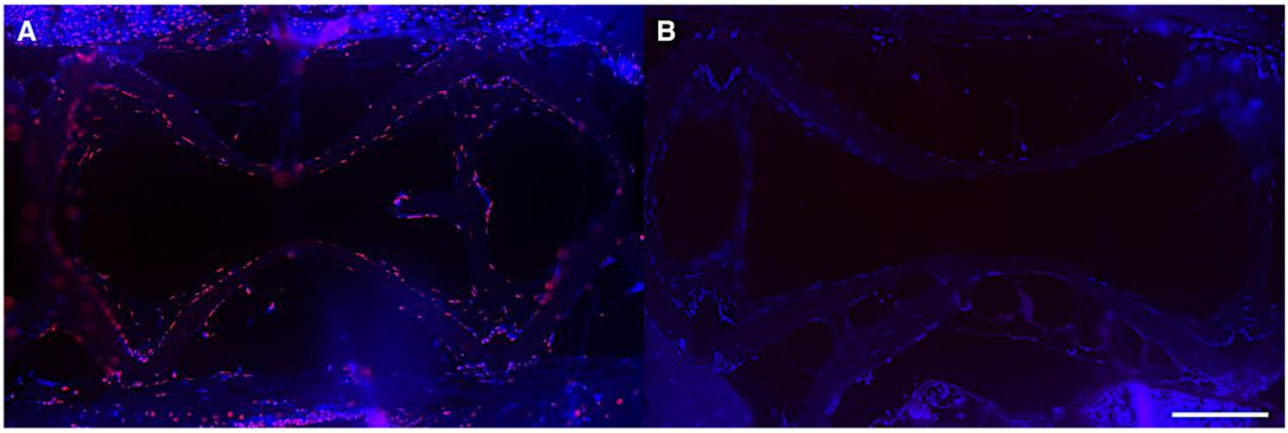

Supplementary Figure 3. TUNEL assay on zebrafish vertebrae. Positive (**A**) and negative (**B**) controls of TUNEL assay on zebrafish vertebrae. TUNEL positive cells are shown by red signal, nuclei are shown by blue signal (DAPI). Scale bar: 100 µm.

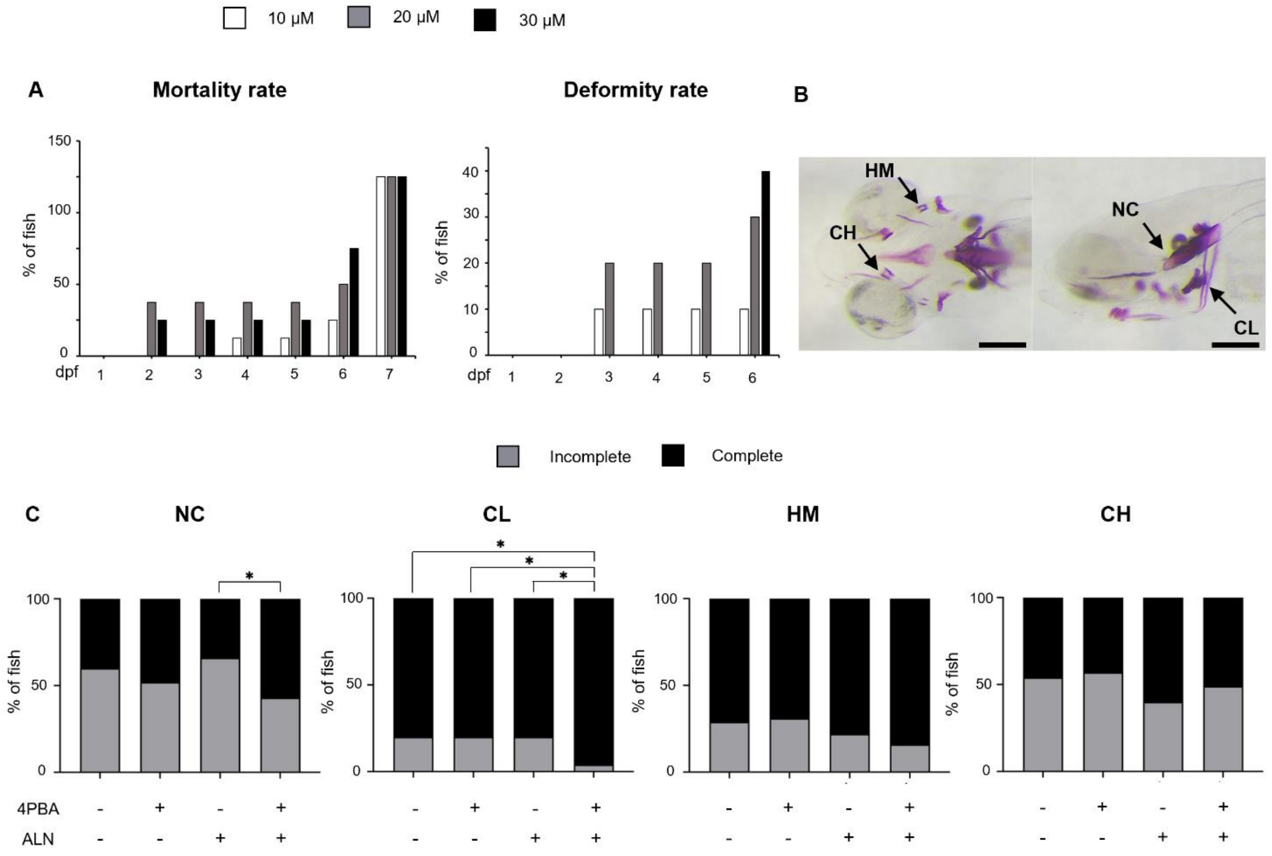

Supplementary Figure 4. ALN toxicity test on WT larvae and 4PBA +ALN treatment on *Chi/+* larvae. (**A**) Mortality and deformity rates in WT larvae treated with 10, 20 and 30 µM concentrations of ALN from 1 to 7 dpf. (**B**) Representative images of 11 dpf zebrafish larvae stained with alizarin red S, allowing mineralized cranial bones detection. Scale bar: 200 µm. (**C**) Level of bone mineralization analyzed in 11 dpf *Chi/+* notochord (NC), cleithrum (CL), hyomandibular (HM) and ceratohyal (CH). * $p < 0.05$.

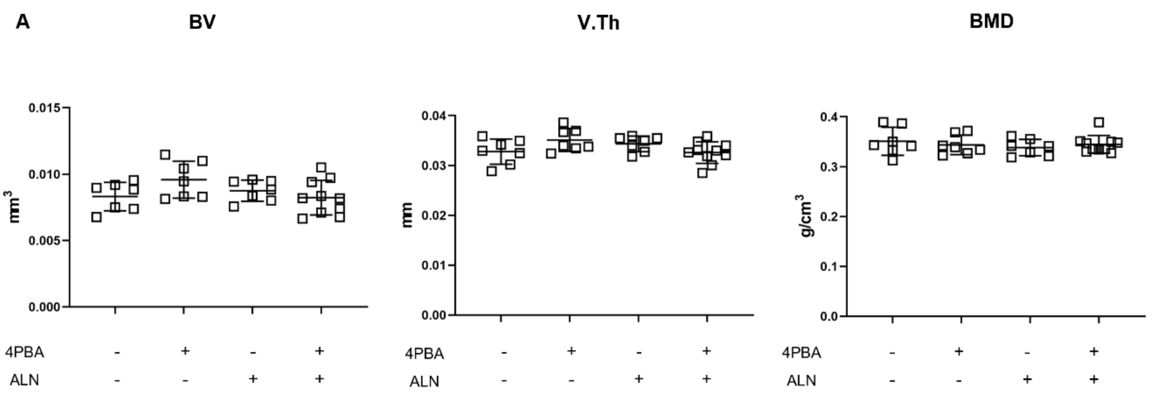

Supplementary Figure 5. Bone geometrical parameters. (**A**) μCT analysis of bone volume (BV), vertebral thickness (V.Th) and bone mineral density (BMD) in WT untreated and treated with 4PBA, ALN and 4PBA+ALN.

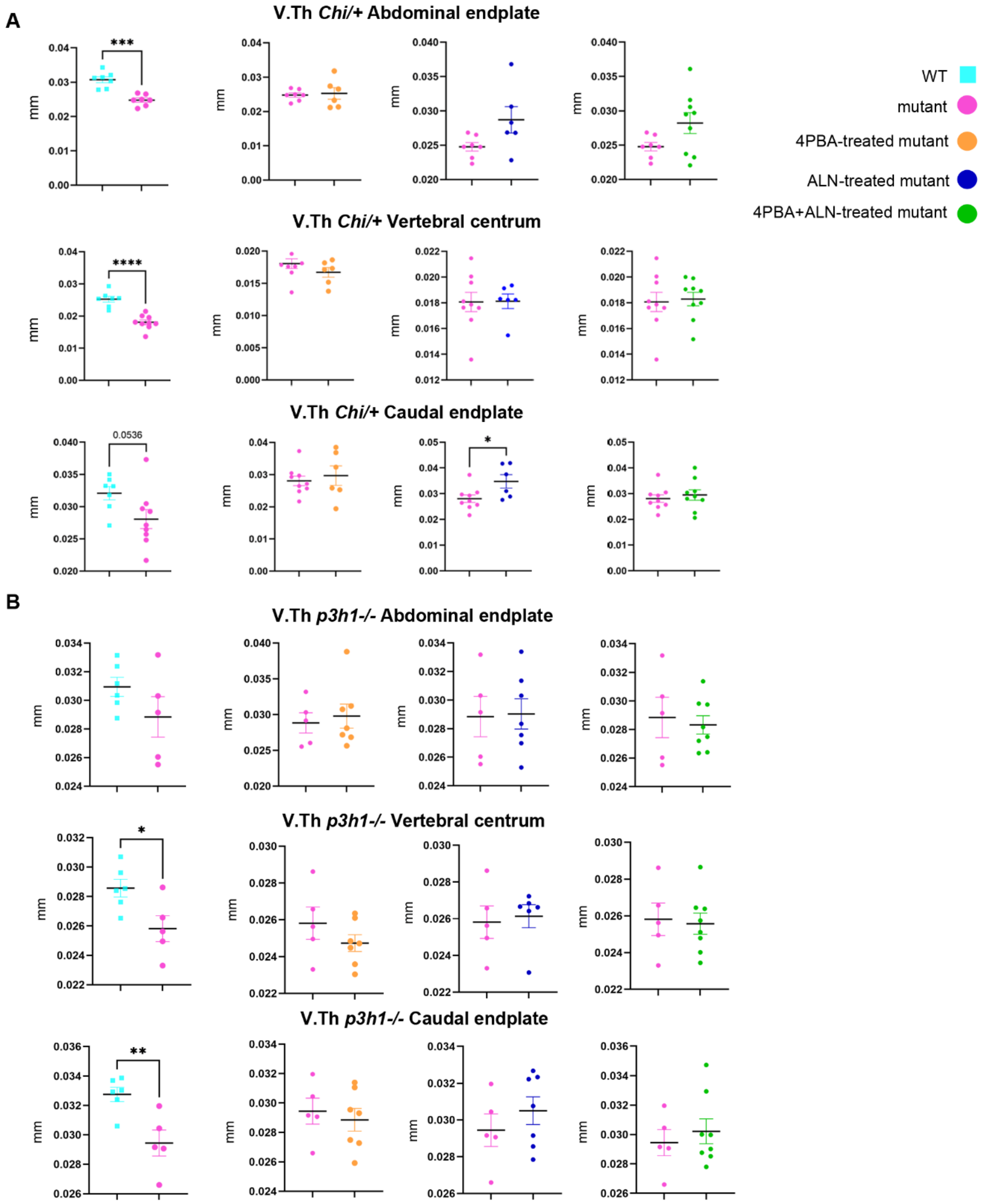

A
V.Th Chi/+ Abdominal endplate
V.Th Chi/+ Vertebral centrum
V.Th Chi/+ Caudal endplate
0.0536
B
V.Th p3h1-/- Abdominal endplate
V.Th p3h1-/- Vertebral centrum
V.Th p3h1-/- Caudal endplate
mm
WT
mutant
4PBA-treated mutant
ALN-treated mutant
4PBA+ALN-treated mutant

Supplementary Figure 6. Graphical representation of 2D thickness distribution in vertebral centrum and endplates. (**A**) Comparison between untreated WT and *Chi/+* and between untreated and treated *Chi/+*. (**B**) Comparison between untreated WT and $p3h1^{-/-}$ and between untreated and treated $p3h1^{-/-}$. Each dots represents the mean of the 2D vertebral thickness of a single fish. * $P < 0.05$, ** $P < 0.01$, ***$P < 0.001$, **** $P < 0.0001$.

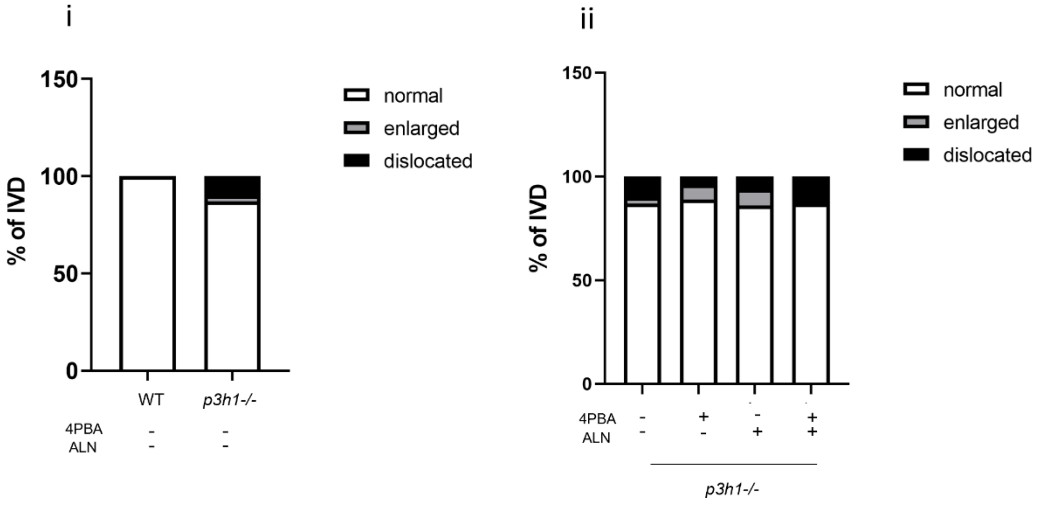

Supplementary Figure 7. ***p3h1***$^{-/-}$ **intervertebral disc (IVD) structure evaluation**. Intervertebral disc (IVD) structure evaluation of untreated WT, *p3h1*$^{-/-}$ (i) and *p3h1*$^{-/-}$ untreated and treated (ii). The percentage of normal, enlarged or dislocated IVD on total number of analyzed IVD is reported.

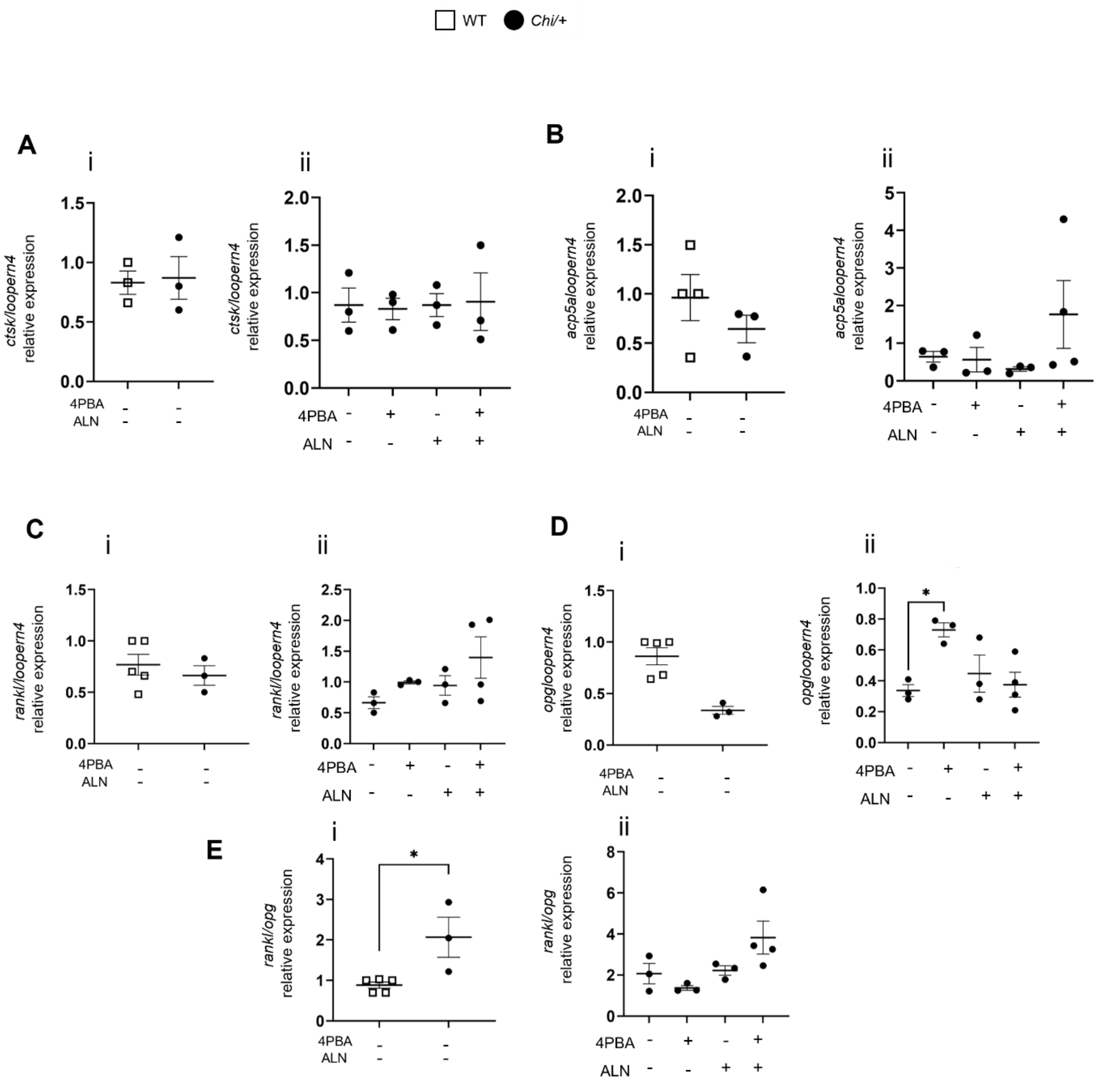

Supplementary Figure 8 | Analysis of osteoclast markers expression in *Chi/*+. RT-qPCR analysis of (**A**) *ctsk* , (**B**) *acp5a,* (**C**) *rankl,* (**D**) *opg,* (**E**) *rankl/opg* in WT, *Chi/*+ (i) and untreated and treated *Chi/*+(ii). Each dot represents a single value. * $p < 0.05$.

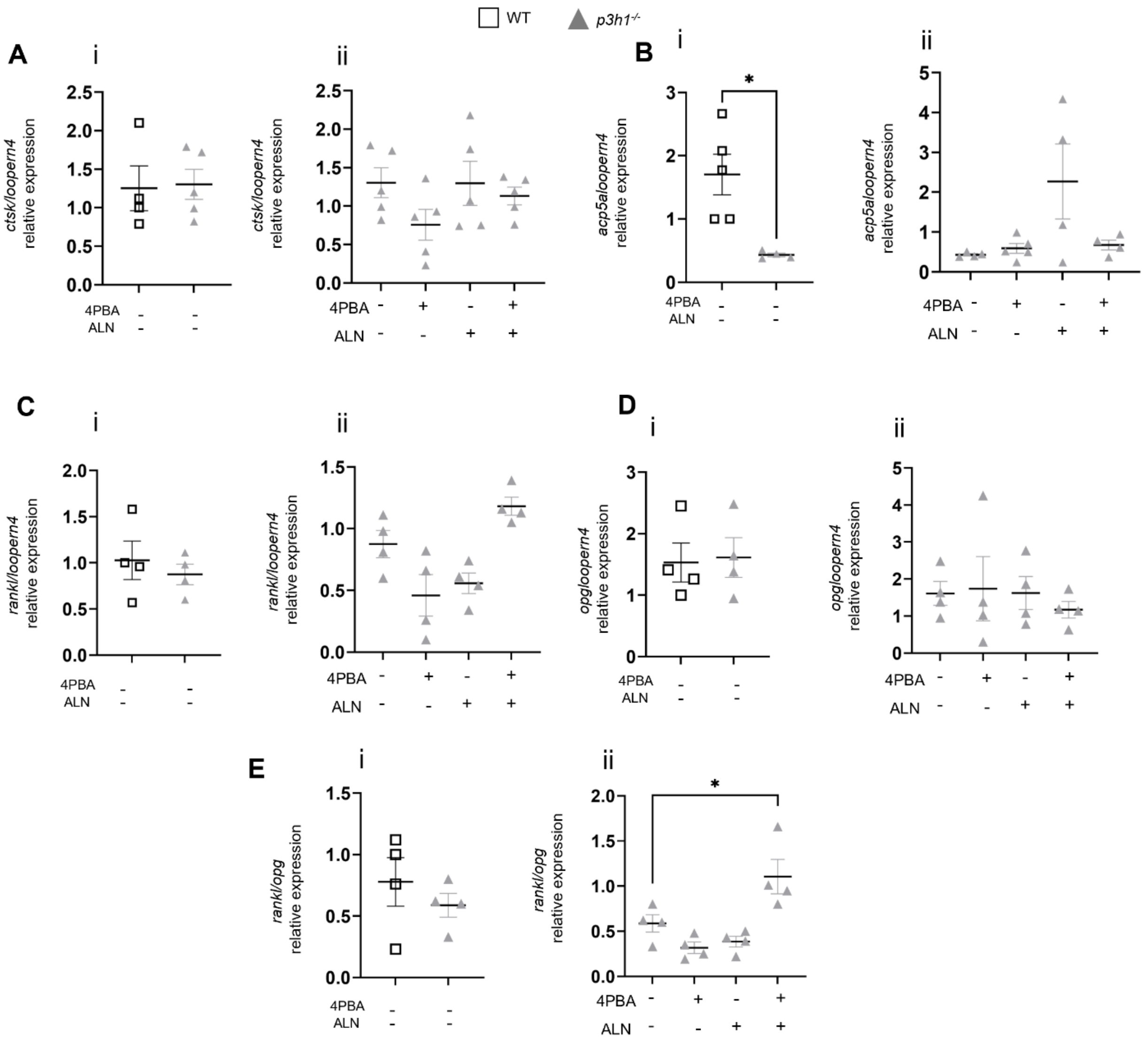

Supplementary Figure 9. Analysis of osteoclast markers expression in *p3h1-/-*. RT-qPCR analysis of (**A**) *ctsk* , (**B**) *acp5a,* (**C**) *rankl,* (**D**) *opg,* (**E**) *rankl/opg* in untreated WT, *p3h1-/-* (i) and untreated and treated *p3h1-/-* (ii). Each dot represents a single value. * $p < 0.05$.

Supplementary materials references

(1) Blum, N.; Begemann, G. Osteoblast de- and redifferentiation are controlled by a dynamic response to retinoic acid during zebrafish fin regeneration. *Development* **2015**, *142* (17), 2894-2903. DOI: 10.1242/dev.120204.
(2) Malbouyres, M.; Guiraud, A.; Lefrançois, C.; Salamito, M.; Nauroy, P.; Bernard, L.; Sohm, F.; Allard, B.; Ruggiero, F. Lack of the myotendinous junction marker col22a1 results in posture and locomotion disabilities in zebrafish. *Matrix Biol* **2022**, *109*, 1-18. DOI: 10.1016/j.matbio.2022.03.002.
(3) Vanhauwaert, S.; Van Peer, G.; Rihani, A.; Janssens, E.; Rondou, P.; Lefever, S.; De Paepe, A.; Coucke, P. J.; Speleman, F.; Vandesompele, J.; et al. Expressed repeat elements improve RT-qPCR normalization across a wide range of zebrafish gene expression studies. *PLoS One* **2014**, *9* (10), e109091. DOI: 10.1371/journal.pone.0109091.
(4) Daponte, V.; Tonelli, F.; Masiero, C.; Syx, D.; Exbrayat-Héritier, C.; Biggiogera, M.; Willaert, A.; Rossi, A.; Coucke, P. J.; Ruggiero, F.; et al. Cell differentiation and matrix organization are differentially affected during bone formation in osteogenesis imperfecta zebrafish models with different genetic defects impacting collagen type I structure. *Matrix Biol* **2023**. DOI: 10.1016/j.matbio.2023.06.003.
